# Psychiatric Manifestation of Anti-LGI1 Encephalitis

**DOI:** 10.3390/brainsci10060375

**Published:** 2020-06-16

**Authors:** Dominique Endres, Harald Prüss, Andrea Dressing, Johanna Schneider, Bernd Feige, Tina Schweizer, Nils Venhoff, Kathrin Nickel, Sophie Meixensberger, Miriam Matysik, Simon J. Maier, Katharina Domschke, Horst Urbach, Philipp T. Meyer, Ludger Tebartz van Elst

**Affiliations:** 1 Section for Experimental Neuropsychiatry, Department of Psychiatry and Psychotherapy, Medical Center, Faculty of Medicine, University of Freiburg, 79104 Freiburg, Germany; bernd.feige@uniklinik-freiburg.de (B.F.); kathrin.nickel@uniklinik-freiburg.de (K.N.); sophie.marie.meixensberger@uniklinik-freiburg.de (S.M.); miriam.matysik@uniklinik-freiburg.de (M.M.); simon.maier@uniklinik-freiburg.de (S.J.M.); tebartzvanelst@uniklinik-freiburg.de (L.T.v.E.); 2 Department of Psychiatry and Psychotherapy, Medical Center, Faculty of Medicine, University of Freiburg, 79104 Freiburg, Germany; tina.schweizer@uniklinik-freiburg.de (T.S.); katharina.domschke@uniklinik-freiburg.de (K.D.); 3 Department of Neurology and Experimental Neurology, Charité, Universitätsmedizin Berlin, 10117 Berlin, Germany; harald.pruess@charite.de; 4 German Center for Neurodegenerative Diseases (DZNE) Berlin, 10117 Berlin, Germany; 5 Clinic of Neurology and Neurophysiology, Medical Center, Faculty of Medicine, University of Freiburg, 79106 Freiburg, Germany; andrea.dressing@uniklinik-freiburg.de; 6 Renal Division, Department of Medicine, Medical Center, Faculty of Medicine, University of Freiburg, 79106 Freiburg, Germany; johanna.schneider@uniklinik-freiburg.de; 7 Department of Rheumatology and Clinical Immunology, Medical Center, Faculty of Medicine, University of Freiburg, 79106 Freiburg, Germany; nils.venhoff@uniklinik-freiburg.de; 8 Center for Basics in Neuromodulation, Faculty of Medicine, University of Freiburg, 79106 Freiburg, Germany; 9 Department of Neuroradiology, Medical Center, Faculty of Medicine, University of Freiburg, 79106 Freiburg, Germany; horst.urbach@uniklinik-freiburg.de; 10 Department of Nuclear Medicine, Medical Center, Faculty of Medicine, University of Freiburg, 79106 Freiburg, Germany; philipp.meyer@uniklinik-freiburg.de

**Keywords:** anti-LGI1 encephalitis, limbic encephalitis, autoimmune psychosis

## Abstract

Background: Anti-leucine-rich glioma-inactivated 1 (LGI1) encephalitis is typically characterized by limbic encephalitis, faciobrachial dystonic seizures and hyponatremia. The frequency with which milder forms of anti-LGI1 encephalitis mimic isolated psychiatric syndromes, such as psychoses, or may lead to dementia if untreated, is largely unknown. Case presentation: Here, the authors present a 50-year-old patient who had suffered from neurocognitive deficits and predominant delusions for over one and a half years. He reported a pronounced feeling of thirst, although he was drinking 10–20 liters of water each day, and he was absolutely convinced that he would die of thirst. Due to insomnia in the last five years, the patient took Z-drugs; later, he also abused alcohol. Two years prior to admission, he developed a status epilepticus which had been interpreted as a withdrawal seizure. In his serum, anti-LGI1 antibodies were repeatedly detected by different independent laboratories. Cerebrospinal fluid analyses revealed slightly increased white blood cell counts and evidence for blood–brain-barrier dysfunction. Magnetic resonance imaging showed hyperintensities mesio-temporally and in the right amygdala. In addition, there was a slight grey–white matter blurring. A cerebral [^18^F] fluorodeoxyglucose positron emission tomography (FDG-PET) examination of his brain showed moderate hypometabolism of the bilateral rostral mesial to medial frontal cortices. Treatment attempts with various psychotropic drugs remained unsuccessful in terms of symptom relief. After the diagnosis of probable chronified anti-LGI1 encephalitis was made, two glucocorticoid pulse treatments were performed, which led to a slight improvement of mood and neurocognitive deficits. Further therapy was not desired by the patient and his legally authorized parents. Conclusion: This case study describes a patient with anti-LGI1 encephalitis in the chronified stage and a predominant long-lasting psychiatric course with atypical symptoms of psychosis and typical neurocognitive deficits. The patient’s poor response to anti-inflammatory drugs was probably due to the delayed start of treatment. This delay in diagnosis and treatment may also have led to the FDG-PET findings, which were compatible with frontotemporal dementia (“state of damage”). In similar future cases, newly occurring epileptic seizures associated with psychiatric symptoms should trigger investigations for possible autoimmune encephalitis, even in patients with addiction or other pre-existing psychiatric conditions. This should in turn result in rapid organic clarification and—in positive cases—to anti-inflammatory treatment. Early treatment of anti-LGI1 encephalitis during the “inflammatory activity state” is crucial for overall prognosis and may avoid the development of dementia in some cases. Based on this case, the authors advocate the concept—long established in many chronic inflammatory diseases in rheumatology—of distinguishing between an “acute inflammatory state” and a “state of organ damage” in autoimmune psychosis resembling neurodegenerative mechanisms.

## 1. Background

Anti-leucine-rich glioma-inactivated 1 (LGI1) encephalitis is typically found in men over 60 years of age and clinically manifests as limbic encephalitis [1,2,3], with a subacute onset of working memory deficits, seizures, or psychiatric symptoms suggesting an involvement of the limbic system [4]. Early symptoms include subtle focal seizures (autonomic or dyscognitive), found in 66% of patients [5]. Faciobrachial dystonic seizures are characteristic of anti-LGI1 encephalitis and are observed in about half of the patients [5,6]. In the course of the disease, 63% of the patients develop tonic-clonic seizures [5], and 60% develop hyponatremia during acute manifestation [5,7]. Magnetic resonance imaging (MRI) reveals signal hyperintensities in the temporo-mesial cortex in over 60% of the patients [5,8]. In addition, blurring of the supratentorial white matter on T2-weighted images may be seen [8]. Typical [^18^F] fluorodeoxyglucose positron emission tomography (FDG-PET) findings include hypermetabolism of the mesial temporal lobe and striatum, which is often asymmetric, with possible dependence on the patient’s handedness, and sometimes also hypometabolism of cortical areas (in particular, frontal) [9,10,11,12]. Electroencephalography (EEG) shows alterations in over half of the patients: epileptic activity in 31% and focal slowing in 25% [5]. Cerebrospinal fluid (CSF) findings may be inconspicuous or may show an increased protein concentration or slight pleocytosis, both in less than 25% of cases [13]. Cell-based assays of CSF were positive in only 53% of the seropositive patients [5]. However, anti-LGI1 antibody-producing plasma cells can still be detected in such patients, confirming that intrathecal production of pathogenic anti-LGI1 antibodies can occur below the detection threshold of clinical routine assays [14]. A tumor association was found in up to 11% of the cases, with thymomas and lung carcinomas being the most common types [1,5]. Previous therapy experience, most commonly with glucocorticoids, as well as with intravenous immunoglobulins or plasmapheresis, has led to symptom improvement in approximately 80% of cases [5]. The seizures usually improve faster than the cognitive symptoms [5]. 

Rationale: Although behavioral changes such as apathy or disinhibition are observed in 90% of patients [5], the relevance of predominantly psychiatric forms with psychosis or affective syndromes is still unclear. Additionally, whether untreated anti-LGI1 encephalitis can lead to dementia-like syndromes is not definitely clarified. Therefore, the aim of this study is to report an atypical case with predominantly psychiatric symptoms and chronification.

## 2. Case Presentation

**Figure 1:**
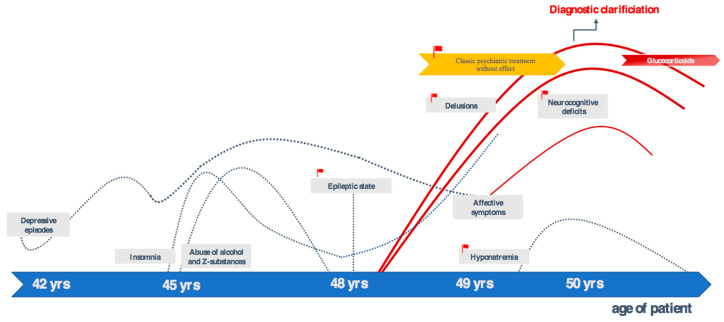
Clinical symptoms and course. Abbreviation: yrs, years.

This paper presents the case of a 50-year-old male German academic who developed neurocognitive deficits, affective symptoms, and predominant delusions for over one and a half years. The patient, as well as his family, have given their signed written informed consent for this case report, including the presented images, to be published. The patient reported a pronounced feeling of thirst, although he was drinking 10–20 liters of water a day, and he was absolutely convinced that he would die of thirst. He did not hear an imperative voice encouraging him to drink more. He said that he needed to drink less water and wondered how his body could retain all the liquid. He was delusionally convinced that he had lost the ability to urinate. In contrast, sonography repeatedly detected no residual urine. Moreover, he suffered from severe insomnia (the patient had the feeling of being completely unable to sleep), reduced cognitive performance—for example, being unable to remember dates—and reduced energy levels, as well as complete loss of interests. In the external assessment, disinhibition, flattened affect, accelerated speech and slow thinking were striking. He had suffered from recurrent depressive episodes since he was 34 years old. For the past eight years, depression had been more severe. In the period between 45 years and 48 years, he had taken high doses of Z-drugs in order to treat insomnia. At the same time, alcohol abuse had developed until two years ago (up to the age of 48 years). At the age of 48, he had suffered a single epileptic status, which was interpreted as the result of alcohol and Z-drug withdrawal in an external hospital. At that time, epileptic activity was described in EEG. Over the last two years he had not consumed any substances (Figure 1). In addition, hyponatremia (i.e., 130 mmol/L; reference range: 136–145 mmol/L) had been revealed half a year prior to diagnosis. On admission to our hospital, a normal sodium concentration was measured. Additional repeated laboratory testing showed a urine osmolality of 73 and 45 mosm/kg, respectively, and a suppressed urine sodium concentration of less than 20 mmol/L. During a thirst trial, urine osmolality increased to 651 mosm/kg, which indicates psychogenic polydipsia and excludes a syndrome of inappropriate antidiuretic hormone (SIADH).

**Figure 2:**
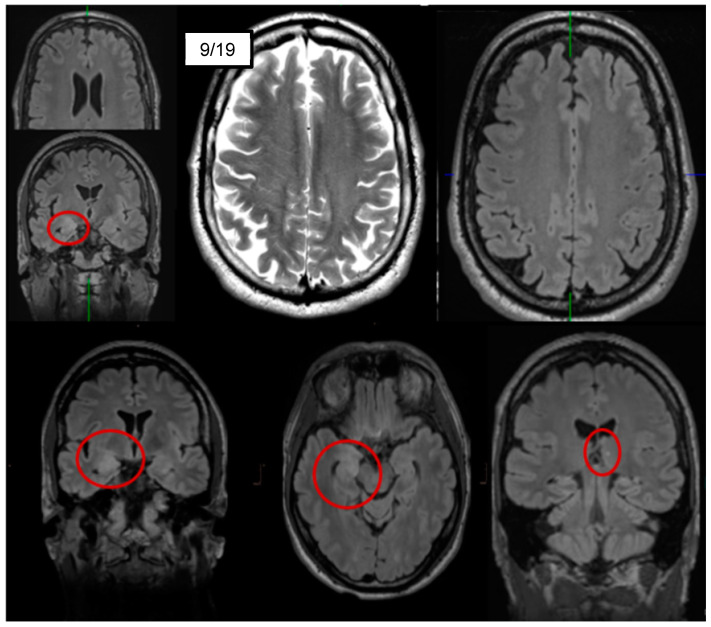
Magnetic resonance imaging showing fluid-attenuated inversion recovery (FLAIR) hyperintensities right-mesio-temporally and on the right side of the corpus amygdaloideum, as well as a small, possibly microangiopathic, lesion in the left thalamus (bottom row). In addition, a slight grey–white matter blurring was observed (top center and right; cf. [8]).

**Figure 3:**
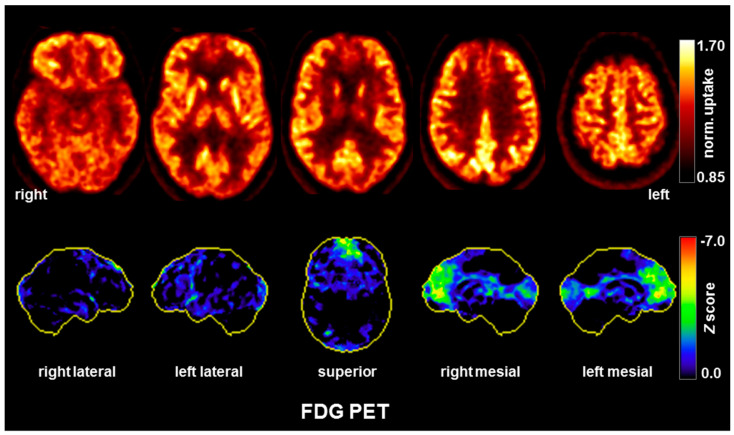
A [^18^F] fluorodeoxyglucose positron emission tomography (FDG-PET) examination revealed moderate hypometabolism of the bilateral mesial to medial frontal cortices. Upper row: transaxial FDG-PET images (voxel-wise FDG uptake normalized to whole brain uptake); lower row: 3D surface projections of regions with decreased FDG uptake (color-coded Z-score, compared to age-matched healthy controls; minor, linear-shaped areas of mild hypometabolism were judged to be non-specific partial volume effects due to atrophy, whereas occipital cortex hypometabolism was probably caused by closing the eyes; [17]). The FDG-PET of the brain was performed 50 min after injection of 214 MBq FDG (Vereos Digital PET/CT, Philips Healthcare, The Netherlands). Abbreviations: R, right; l, left.

Diagnostic findings: The diagnostic examinations were conducted approximately one and a half year after symptom exacerbation after admission to our special ward. In serum, anti-LGI1 antibodies were repeatedly positive in different laboratories (see Table 1), whereas CSF antibody testing was negative. A serum titer of 1:80 (reference <1:20) was measured using cell-based assays. CSF analyses showed normal to slightly elevated white blood cell (WBC) counts (maximum 5/µL; reference <5/µL) and evidence of a blood–brain barrier dysfunction (protein concentration up to 557 mg/L; reference: <450 mg/L; albumin quotients up to 9.5, reference: <8). Oligoclonal bands were always negative. Fluid-attenuated inversion recovery (FLAIR) MRI sequences depicted hyperintensities right-mesio-temporally and on the right side of the amygdala. In the left thalamus, a small, possibly microangiopathic, lesion was detected. In addition, there was a slight grey–white matter blurring (cf. [8] Figure 2). The routine EEG was normal in the visual assessment. Independent components analyses or rather automatic detection of intermittent EEG slowing remained inconspicuous (cf. [15]). A cerebral FDG-PET examination showed moderate hypometabolism of the bilateral mesial to medial frontal cortices, which was interpreted as being possibly due to an early manifestation of frontotemporal dementia [16], whereas a sequela of substance and/or alcohol abuse was rated to be less likely. It should be noted that there was no mesial temporal or striatal hypermetabolism (Figure 3), which can be present in active limbic or anti-LGI1 encephalitis. A whole-body FDG-PET/computer tomography scan detected no metabolic or structural pathologies suggestive of malignancy or inflammation. Neuropsychological testing using the Consortium to Establish a Registry for Alzheimer’s Disease (CERAD) test battery showed deficits in Mini Mental State Examination (25 of maximum 30 points), word-list savings, and trail-making tests B and A/B. The test for attentional performance by TAP showed deficits in working memory (missings, false alarms), set shifting (overall index) and alertness (reaction times with and without sound). All diagnostic findings are summarized in Table 1.

Illness, somatic, and family histories: The patient’s past medical history was inconspicuous in terms of in-utero and birth complications. He had not suffered from febrile convulsions, inflammatory brain diseases, relevant systemic infections, or craniocerebral traumata during his childhood or adolescence. There was no evidence of neurodevelopmental or personality disorder. Seven years earlier, at the age of 43, he had developed a deep vein thrombosis by unclear coagulopathy. For several years, he had suffered from leg ulcer on the left inner ankle. He had also suffered from arterial hypertension, treated with ramipril 10 mg/day. His history of autoimmune diseases, infections, or cancer was negative. In the family history of neuropsychiatric diseases, only his father had suffered from depression.

Treatment and outcome: Classic psychiatric treatment with sertraline, venlafaxine, mirtazapine, reboxetine, several tricyclic drugs, lithium, zopiclone, olanzapine, risperidone, haloperidol, quetiapine, and clozapine previously administered had no relevant positive effect on symptom relief. Under clozapine, he had developed myocarditis. After immunological findings gave evidence for chronified limbic autoimmune encephalitis, glucocorticoid pulse therapy with 500 mg of intravenous methylprednisolone daily for five days and subsequent oral tapering over two months (starting with 50 mg) led to slight improvement of mood and neurocognitive symptoms including temporal orientation, executive functions, word list savings and alertness times (with no difference between the conditions with and without sound). However, new deficits were found in fluency and word list learning with more word intrusions, while deficits in working memory and set shifting were persisting. A second steroid pulse 3 months after the first pulse with oral tapering did not lead to relevant improvement. However, the signal hyperintensities in the MRI showed a tendency to reduction. Further treatment (e.g., with plasmapheresis and/or rituximab) was refused by the patient and his legally authorized parents. Furthermore, l-thyroxine (due to hypothyroidism), vitamin B1 (prophylactic after earlier alcohol abuse), and vitamin D (due to serologically proven deficiency) were supplemented.

## 3. Discussion

This case report describes a patient with chronified anti-LGI1 encephalitis who had exhibited neurocognitive, affective, and predominant delusional symptoms for at least one and a half years, as well as pre-existing insomnia, addiction, and depression.

### 3.1. Diagnostic Assessment

The anti-LGI1 antibody finding, in combination with the CSF alterations and the mesio-temporal hyperintensities and slight grey–white matter blurring detected on MRI [8] led to the diagnosis of anti-LGI1 encephalitis. The inconspicuous EEG findings in the presented case are rare in this context [5]. Importantly, there was no mesial temporal or striatal hypermetabolism on FDG-PET, which would typically be expected in active limbic encephalitis during the “inflammatory activity state” [9,12,18,19,20,21]. However, occasional mesial frontal involvement has also been described in anti-LGI1 encephalitis, including both hypermetabolism [12] and hypometabolism [10,11,19]. We therefore assumed the chronic stage (“damage stage”) of anti-LGI1 encephalitis, in which neurodegeneration and hypometabolism may occur.

The isolated psychiatric manifestation over the course of at least one and a half years without epileptic seizures, predominant delusions, and affective flattening, which could have led to a diagnosis of schizophrenia, is exceptional. The neurocognitive symptoms, however, were typical for patients with anti-LGI1 encephalitis and are observed in 97% of cases [5]. It can be assumed that the incipient anti-LGI1 encephalitis had contributed to the occurrence of status epilepticus two years earlier. The striking EEG finding at that time might also have corresponded to anti-LGI1 encephalitis [5], although no antibody testing was carried out at that time to evaluate the putative autoimmune genesis. Less likely, it can be speculated that Z-drug abuse was an early form of self-treatment for insomnia and might have lowered the seizure threshold caused by the anti-LGI1 antibodies. As is well known, anti-LGI1 antibodies are associated with severe insomnia, and the disruption of the LGI1 protein can trigger temporal seizures [5]. Hyponatremia, a hallmark of anti-LGI1 encephalitis, was initially observed. It was reported in 60% of anti-LGI1 encephalitis patients [1], although it is typically discussed in the context of SIADH. However, while psychogenic polydipsia was evident, our patient showed no signs of SIADH. The mechanisms contributing to hyponatremia in patients with anti-LGI1 encephalitis have been investigated in a very limited number of patients [22,23], and additional mechanisms, including psychogenic polydipsia, may promote hyponatremia in the context of anti-LGI1 encephalitis. LGI1 is expressed in the renal tubular system [24], which provides another potential mechanism for the dysregulation of the sodium balance. In the presented case, it remained unclear whether the strong feeling of thirst the patient reported had resulted from a primary organic cause (i.e., mild inflammation along the hypothalamic–pituitary neuraxis) or was a secondary delusional phenomenon [22]. 

### 3.2. Limitations

The hypothyroid metabolic state may have contributed to the development of the depressive symptoms in the previous history. Over the course of time, however, l-thyroxine was substituted and led to normal thyroid hormone levels. The extent to which earlier alcohol consumption had contributed to the observed cognitive dysfunction and alterations detected by FDG-PET was not entirely clear, but it might have accelerated the cognitive deficits. Nevertheless, in the authors’ experience, the presentation of chronic substance and/or alcohol abuse causes more widespread rather than circumscribed metabolic deficits on FDG-PET. Typical signs of Wernicke’s encephalopathy, such as ocular abnormalities, gait disturbance, and trunk ataxia [25], were not present; moreover, the anti-LGI1 antibody findings and mesio-temporal MRI changes clearly could not be attributed to this. From our perspective, these somatic findings, as well as the clinical course (e.g., with status epilepticus), argue against the beginning of classical frontotemporal dementia, which could have been assumed from the FDG-PET findings [16]. The anti-inflammatory treatment resulted in only a slight improvement of mood; however, further anti-inflammatory treatment with plasmapheresis or rituximab—under which further improvement could possibly occur—was unfortunately rejected by the patient and his legally authorized parents. The rejection of anti-inflammatory therapy in such constellations brings with it new ethical questions regarding compulsory treatment in psychiatry [26]. Previous research has reported the benefits of acetylcholinesterase inhibitors (e.g., rivastigmine) to ameliorate rapidly progressive organic dementia [27]. A treatment with acetylcholinesterase inhibitors had not been attempted in the presented patient at the time of publication.

### 3.3. Clinical Consequences

The presented case shows that predominantly psychotic syndromes do exist in the context of anti-LGI1 encephalitis. A similar case of a young patient with anti-LGI1 encephalitis with psychotic symptoms and memory deficits but also with typical faciobrachial seizures was recently published [28]. The term “autoimmune psychosis” was recently suggested for such cases, and red flags and consensus criteria have been defined [29,30,31,32,33,34]. The red flags for underlying autoimmune encephalitis in this case were polymorphic psychiatric symptoms with severe cognitive deficits, the atypical age for psychotic development, the aggravation of symptoms despite psychopharmacological treatment, and, probably, even the singular status epilepticus two years earlier (cf. [32]). The patient’s poor response to anti-inflammatory drugs may be due to the delayed start of anti-inflammatory treatment, since it is well known that early treatment is associated with better outcomes in autoimmune encephalitis [35]. Thus, it could be assumed that untreated autoimmune limbic encephalitis turned into a frontotemporal dementia-like syndrome with hypometabolic changes on FDG-PET, which has been suggested already in the era before anti-LGI1 antibody testing became available [36]. 

### 3.4. Conceptual Considerations

From the authors’ point of view, this case demonstrates the difference, already established in many chronic inflammatory diseases in rheumatology, between an “acute inflammatory state” and a “state of organ damage.” In the “acute inflammatory state,” immunosuppressive therapy can be successfully carried out to control or completely suppress the disease activity and thus possibly avoid irreversible damage. In a “state of organ damage,” established organ damage associated with organ dysfunction has already occurred due to inflammatory activity. In this condition, patients with chronic inflammatory rheumatic diseases can sometimes have severe symptoms due to organ dysfunction (e.g., renal insufficiency, myocardial scarring, or irreversible paralysis), but inflammatory activity may no longer be apparent. Accordingly, anti-inflammatory therapy will cease to be effective and may even place patients at further risk. In the case of vasculitis, scores already exist (e.g., Birmingham Vasculitis Activity Score for activity and the Vasculitis Activity Index for damage; [37]). In Sjögren’s syndrome, a distinction is made between an “acute inflammatory state” with lymphoproliferative inflammation, which can be successfully influenced therapeutically, and the chronic phase of organ damage, in which Sicca symptoms become established due to the complete destruction of the salivary and lacrimal glands, which can no longer be influenced by immunosuppression. In the presented case, the normalized EEG findings, and hypometabolism on FDG-PET, as well as the poor response to immunosuppressive treatment, may well indicate the presence of a “state of organ damage.” Such residual states of formerly active inflammations have occasionally also been proposed for other forms of encephalitis (e.g., a patient with anti-Ri limbic encephalitis and prominent mesiotemporal hypometabolism; [9]). Future studies should focus on detecting “activity markers” in the “acute inflammatory state” and signs of irreversible brain damage in the “damage state” in autoimmune encephalitis/psychosis. This could help to identify patients who could benefit from immunosuppressive treatment, even during longer disease courses, as such treatment is described in patients with autoimmune encephalitis even 21 months after symptom onset [38]. At the same time, frustrating and potentially harmful anti-inflammatory treatments could be avoided.

## 4. Conclusions

Predominantly neurocognitive and psychotic disorders may be caused by milder forms of anti-LGI1 encephalitis, even in patients with addiction or other pre-existing psychiatric conditions. The detection of red flags for the presence of autoimmune encephalitis, such as epileptic seizures, hyponatremia, and atypical age of psychosis onset, should result in rapid clarification of organic causes and—in positive cases—in anti-inflammatory/B cell-depleting treatment during the “acute inflammatory state”. Early treatment of autoimmune limbic encephalitis is crucial for a favorable prognosis. However, untreated limbic encephalitis may well evolve into chronified frontotemporal dementing syndromes reflecting “a state of cerebral organ damage”.

## Figures and Tables

**Table 1:** Diagnostic findings approximately 1.5 years after symptom exacerbation. Abbreviation: FDG-PET, [^18^F] fluorodeoxyglucose positron emission tomography.

**Neuropsychiatric examination**	**Severe formal thought disorder and attention, concentration and memory deficits, flattened mood. Strongly reduced energy level and apathy. Abulia, loss of interests, dramatically reduced activity, delusions, severe insomnia, disinhibition**. No hallucinations, no suicidal tendencies, normal appetite. **Scanning speech, mild dysarthria, at the upper extremity slightly left-emphasized muscle reflexes, patellar tendon reflex with widened reflex zone on both sides.** In addition, inconspicuous neurological examination.
**Blood analyses**	Blood cell count, liver/kidney/pancreas values, and C-reactive protein were normal. **Calcium levels were intermediately discreetly elevated and normalized themselves in the course of time**. **Hyponatremia in the prehistory (i.e., 130 mmol/L a half year before; reference range: 136–145 mmol/L)**, during the clarification as an inpatient the sodium values were normal. Folic acid and Vitamin B12 levels were normal. **Selenium (52 µg/L; reference: 75–140 µg/L) and Vitamin D (9.9 ng/mL; optimal: >30 ng/mL) were reduced**. Thyroid-stimulating hormone, triiodothyronine, and thyroxine levels were in normal ranges. Autoantibodies against thyroglobulin, TSH receptor and thyroid peroxidase were not detectable. Antibody testing for Lyme borreliosis, syphilis and HIV were negative. **Toxoplasmosis IgG antibodies were positive** (IgM antibodies and PCR in CSF were negative). The Bartonella henselae and Hepatisis B/C/E serologies were negative. A quantiferon test was also negative. No IgG autoantibodies against the following intracellular onconeural antigens Yo, Hu, CV2/CRMP5, Ri, Ma1/2, SOX1, Tr, Zic4 or the intracellular synaptic antigens GAD65/amphiphysin were found using Ravo line assay®. No IgG autoantibodies against the following intracellular onconeural antigens—amphiphysin, CV2/CRMP5, Ma2/Ta, Ri, Yo, Hu, recoverin, Sox1, Titin, Zic4 and DNER/Tr—were found using the immunoblot method (laboratory Krone, Bad Salzufflen, Germany). **In the serum, anti-LGI1 IgG autoantibodies were repeatedly slightly positive (using fixed cell biochip assays from Euroimmun^®^). A titer of 1:80 (reference <1:20) was discovered using cell-based assays (laboratory Krone, Bad Salzufflen, Germany)**. RIA showed no increased anti-VGKC autoantibody levels. IgG autoantibodies against other neuronal cell surface antigens (NMDA-R, AMPA-1/2-R, GABA_B_-R, DPPX, CASPR2) were negative (using fixed cell biochip assays from Euroimmun^®^). No IgG autoantibodies against different other neuronal cell surface antigens (GAD65, NMDAR, GABA_B_-R, IgLON5, AMPAR2, DPPX, CASPR2, Glycin-R, mGluR5/1) were found using cell-based assays (laboratory Krone, Bad Salzufflen, Germany). Aquaporin 4 and MOG antibodies were negative. **“Tissue-based assay” using indirect immunofluorescence on unfixed murine brain tissue (Prof. Prüss, Berlin) showed an unspecific nuclear pattern of IgG binding**. Screening for serum antinuclear antibodies using indirect immunofluorescence on HEp-2000® cells was negative. Anti-neutrophil cytoplasmic, antiphospholipid, and anti-mitochondrial antibodies were negative. Rheumatoid factor was negative. Analyses of the complement system (C3, C4, CH50 and C3d) showed no relevant findings. Traceable evidence of a Bence Jones proteinuria of the Kappa type. The kappa and lambda chains in the serum were negative.
**Cerebrospinal fluid analyses (was performed three times after in-patient admission)**	**Normal to slightly increased white blood cell counts (3–5/µL; reference <5/µL).** **Normal to slightly increased protein concentrations (362–569 mg/L; reference <450 mg/L).** **Normal to slightly increased age-corrected albumin quotients (6.4–9.5; reference: <8).** No CSF specific oligoclonal bands; IgG index not increased. No intrathecal IgG, IgM or IgA synthesis. No IgG autoantibodies against the following intracellular onconeural antigens—amphiphysin, CV2/CRMP5, Ma2/Ta, Ri, Yo, Hu, recoverin, Sox1, Titin, Zic4, DNER/Tr—were found using the immunoblot method (laboratory Krone, Bad Salzufflen, Germany). IgG autoantibodies against neuronal cell surface antigens (NMDA-R, AMPA-1/2-R, GABA_B_-R, DPPX, LGI1, CASPR2) were negative (using fixed cell biochip assays from Euroimmun^®^). No IgG autoantibodies against different neuronal cell surface antigens (GAD65, NMDAR, GABA_B_-R, IgLON5, AMPAR2, DPPX, LGI1, CASPR2, Glycin-R, mGluR5/1) were found using cell-based assays (laboratory Krone, Bad Salzufflen, Germany). RIA showed no increased anti-VGKC antibody levels. **“Tissue-based assay” using indirect immunofluorescence on unfixed murine brain tissue (Prof. Prüss, Berlin) showed antibodies against many blood vessels of different sizes, but with relatively low signal.** 14-3-3 protein was not increased.
**Cerebral magnetic resonance imaging**	**Right-sided FLAIR hyperintensities mesio-temporally and in the amygdala without contrast enhancement.** **In the left thalamus, a small, possibly microangiopathic lesion was detected.** **Discrete grey–white matter blurring.**
**Electroencephalo-graphy**	Normal alpha-electroencephalography. No slow wave activity. No epileptic activity. No dysrhythmia. **Two years ago, after status epilepticus, epileptic activity was reported.**
**FDG-PET**	**Moderate hypometabolism of the bilateral mesial to medial frontal cortices.** No lesions or metabolic changes suspicious of malignancy on whole-body FDG-PET/computer tomography.
**Heart examination**	Inconspicuous resting electrocardiography (**but intermittent T-wave changes during clozapine-induced myocarditis**). Transthoracic echocardiography showed normal findings (**but intermittent left-ventricular hypokinesia during clozapine induced myocarditis**). In long-term electrocardiography, continuous normocardial to **tachycardial** sinus rhythm.
**Computer tomography and x-ray thorax**	**Smaller nodulus in the anterior left lower lobe**. No evidence of malignant thoraco-abdominal lesions in **left diaphragm elevation. Triangularly configured compression in the apicoventral lower lobe primarily non-specifically scarred.**
**Bronchoscopy**	**Slight acute tracheobronchitis.In the biopsy, signs of chronic bronchitis with submucosal fibrosis and deep hyaline bronchial cartilage as well as only very sparse alveolar parenchyma were detected**. No evidence of malignancy. No growth of legionella.
**Sonography of the abdomen**	**Small cyst in the liver.** **On the left, rather large kidney, three cysts.**
**Electromyography**	**Fasciculations of the legs**, which can occur in the context of polyneuropathy, neuromyotonia or also as “benign fasciculations”.

Altered findings are written in bold.
